# Supplementary material for: Increase in metazoan ecosystem engineering prior to the Ediacaran–Cambrian boundary in the Nama Group, Namibia
Source: R Soc Open Sci. 2019 Sep 25;6(9):190548. doi: 10.1098/rsos.190548 (PMC6774933; doi:10.1098/rsos.190548)
Supplement: Supplementary material for: Increase in metazoan ecosystem engineering prior to the Ediacaran-Cambrian boundary in the Nama Group, Namibia [file rsos190548supp1.docx]

**Supplemental Material for “Increase in metazoan ecosystem engineering prior to the Ediacaran-Cambrian boundary in the Nama Group, Namibia”**

**S1.** The following are descriptions of ichnotaxa present in the Nama Group trace fossil record:

Plug-shaped burrows (Figure 2E, F, G) – shallow (<1 cm) vertical burrows 2-4mm in diameter, and preserved on the top-surfaces of slabs in both positive and negative relief. In the Zaris Subbasin these are found in the Vingerbreek and Neiderhagen Members, while in the Witputs subbasin they are found in the Kliphoek, Nasep, and Spitzkop Members. When examined in cross section these burrows either exhibit chevron cone-in-cone infill similar to *Conichnus* isp., or lack any layered infill, and thus more similar to *Bergaueria*. Plug-shaped burrows tend to be statistically paired, with neighboring burrows consistently 4-5 mm apart on the bedding plane (S3), but in cross section no evidence was found for U-shaped structures joining the two. Similar traces have been previously described from the Nama Group (Darroch et al., 2016) as well as from the Lower Cambrian Wood Canyon Formation in Death Valley (Mata et al., 2012). We note that some trace fossils classified as plug-shaped burrows may represent a poorly preserved variant of *Treptichnus* (Jensen et al., 2000), which can be distinguished by cutting open and polishing the samples. However, when samples were not able to be cut open, we conservatively describe them as plug-shaped burrows.

*Helminthoidichnites* (Fitch, 1850; Figure 2A). – Small (1-3 mm), curvilinear and unbranching horizontal trace fossils. We find these *Helminthoidichnites* in short, sinuous forms in the Nasep Member of the Witputs Subbasin, where there is a general angle of curvature on the length of the burrow path until termination, and in larger looping forms in the Urusis Member of the Zaris subbasin.

*Helminthopsis* (Heer, 1877; Figure 2B, C). – Meandering, horizontal, and unbranched burrows 1-2 mm in width and frequently >10 cm long. *Helminthopsis* appears in the Witputs Subbasin Spitzkop Member. The burrows occasionally disappear along the burrow path, but it is unclear whether this is due to incomplete preservation or some vertical movement into the substrate (see Carbone & Narbonne, 2014; Tarhan et al., 2017). We find these preserved as small sinuous traces found in the Nasep Member (Witputs Subbasin). These *Helminthopsis* are around 0.5 mm in width and 1 cm length, but are more commonly found exhibiting shorter burrow path lengths. They are found on bedding planes in close proximity to larger trace fossils.

*Parapsammichnites* (Buatois et al., 2018; Figure 2H). – Large, horizontal burrows which maintain a consistent diameter of ~1 cm, and which extend for many 10s cm on the bedding plane (Figure 2). *Parapsammichnites* appear exclusively in the Witputs Subbasin Spitzkop Member. The trace fossils exhibit relatively high relief on the bedding plane, and meander in highly curved patterns, often cross-cutting themselves and/or other traces. *Parapsammichnites* preserves a backfill pattern in the burrows, indicating a sediment ‘bulldozing’ behavior (Buatois et al., 2018).

*Planolites* (Nicholson, 1873; Figure 2D). – Simple, curving unlined burrows horizontal to the bedding plane. *Planolites* was found in the Spitzkop Member at Farm Swartpunt and Camp Koelkrans (Witputs subbasin). *Planolites* is preserved in positive relief, maintains a consistent burrow diameter and tends to cross-cut other *Planolites* trace fossils.

*Treptichnus* (Miller, 1889; Figure 2I, J, K, L). – Shallow, upward-curving burrow segments which connect in zig-zag patterns in straight to looping burrows (see also Wilson et al., 2012). Treptichnids first appear in the Nasep Member in the Witputs Subbasin at the Farm Arimas and Canyon Roadhouse localities, where burrows are characterize by short, 3-5mm long segments which form a gently curved, rope-like path (Figure I). The traces are made up of 3 or 4 segments that maintain a constant width of around 3 mm throughout the entire trace. The combined segments reach lengths of 1-2 cm. The segments which make up each trace are all consistently arranged on one side of the middle of the trace (Figure 2). Nasep strongly resemble the treptichnids from the base of the Huns member which were previously described by Jensen et al. (2000).

At the Farm Swartpunt locality in the Spitzkop Member of the Witputs Subbasin, *Treptichnus* appears as burrows with frequent Y-shaped branches which diverge at a consistent angle of 55-60° (Figure J). The burrows maintain a consistent diameter of around 1mm. We note that due to the regular angle of branching, it is unlikely that these branching *Treptichnus* are a result of the intersection of different imcompletely preserved horizontal burrows, and instead or more likely due to the trace-maker’s successive probing movements (Bromley, 1996). These trace fossils resemble other Ediacaran and early Cambrian *Treptichnus*-type traces from the Mackenzie Mountains in Northwest Canada (Carbone & Narbonne, 2014).

*Treptichnus* also occurs at the Camp Koelkrans locality in Spitzkop member in the thin fine-grain sandstones of interbedded sandstone-carbonate facies. Here, *Treptichnus* is preserved as a positive relief small circular to oval shaped segments which connect below the surface, forming a C-shaped loop (Figure K). These *Treptichnus* trace fossils are found in a dense assemblage with plug-shaped burrows, simple horizontal traces (*?Helminthopsis*), and shallow U-shaped burrows (*?Arenicolites*).

Ichnospecies *Treptichnus* *pedum* (Seilacher, 1955; Figure L). – Large, cm-scale trace fossils comprised of conjoined segments in a looping shape, preserved due to the successive probing movement of the trace-maker. *T. pedum* occurs at the Farm Sontaagsbrun locality in the Cambrian Nomtsas Member (Figure 2L). *T. pedum* does not occur with any other trace fosils. We note that although it is not reported in this dataset,

**S2.** Fossil database, including sample names, assigned ichnotaxon, and localities where collected with corresponding stratigraphic member. The following database was constructed from field work which took course during mid-July to mid-August 2016, and August 2017. Approximately two to three days were spent at each locality.

| **Trace fossil slab sample name** | **farm** | **Present trace fossils** | **Stratigraphic member/formation** | **Subbasin** |
| --- | --- | --- | --- | --- |
| 17-FR-1.01 | Koelkrans | Plug-shaped, *Treptichnus* | Spitzkop | Witputs |
| 17-FR-1.04 | Koelkrans | *Planolites* | Spitzkop | Witputs |
| 17-FR-1.07 | Koelkrans | *Planolites,* Plug-shaped | Spitzkop | Witputs |
| 17-FR-2.3 | Koelkrans | Plug-shaped | Spitzkop | Witputs |
| 17-FR-CK-1 | Koelkrans | *Parapsammichnites* | Spitzkop | Witputs |
| 17-FR-CK-2 | Koelkrans | *Parapsammichnites* | Spitzkop | Witputs |
| 17-FR-CK-3 | Koelkrans | *Parapsammichnites* | Spitzkop | Witputs |
| 17-FR-CK-4 | Koelkrans | *Parapsammichnites* | Spitzkop | Witputs |
| 17-FR-CK-5 | Koelkrans | *Parapsammichnites* | Spitzkop | Witputs |
| 2016-BH-1.11 | Berghoek | Plug-shaped | Vingerbreek | Zaris |
| 2016-BH-1.13 | Berghoek | Plug-shaped | Vingerbreek | Zaris |
| 2016-BH-1.14 | Berghoek | Plug-shaped | Vingerbreek | Zaris |
| 2016-BH-x.1 | Berghoek | Plug-shaped | Vingerbreek | Zaris |
| 2016-DD-1.1 | Kuibis | Plug-shaped | Kliphoek | Witputs |
| 2016-DD-1.15 | Kuibis | Plug-shaped | Kliphoek | Witputs |
| 2016-DD-1.2 | Kuibis | Plug-shaped | Kliphoek | Witputs |
| 2016-DD-1.5 | Kuibis | Plug-shaped | Kliphoek | Witputs |
| 2016-HB-11.1 | Hansburg | Plug-shaped | Kliphoek | Witputs |
| 2016-HB-pi.1 | Hansburg | Plug-shaped | Kilphoek | Witputs |
| 2016-HH-1.1 a | Haruchas | Plug-shaped | Vingerbreek | Zaris |
| 2016-HH-1.1 b | Haruchas | Plug-shaped | Vingerbreek | Zaris |
| 2016-HH-1.15 | Haruchas | Plug-shaped | Vingerbreek | Zaris |
| 2016-HH-1.2 | Haruchas | Plug-shaped | Vingerbreek | Zaris |
| 2016-HH-1.8 | Haruchas | Plug-shaped | Vingerbreek | Zaris |
| 2016-HH-3.4 | Haruchas | Plug-shaped | Vingerbreek | Zaris |
| 2016-KK-1.4 | Spider Ridge | Plug-shaped | Vingerbreek | Zaris |
| 2016-NR-1.1 | Neuras | Plug-shaped | Neiderhagen | Zaris |
| 2016-SB-1.31 | Sontaagsbrun | *Treptichnus* | Nomtsas | Witputs |
| 2016-SB-2.31 | Sontaagsbrun | *Treptichnus* | Nomtsas | Witputs |
| 2016-SB-float-pedum | Sontaagsbrun | Treptichnus | Nomtsas | Witputs |
| 2016-US-10.1 | Urusis | *Helminthoidichnites* | Urusis | Zaris |
| 2017-AR-1.04 | Arimas | *Helminthopsis* | Nasep | Witputs |
| 2017-AR-1.05 | Arimas | *Helminthopsis* | Nasep | Witputs |
| 2017-AR-1.15 | Arimas | *Helminthopsis, Helminthoidichnites* | Nasep | Witputs |
| 2017-CR-1.04 | Canyon_Roadhouse | *Helminthoidichnites* | Nasep | Witputs |
| 2017-CR-1.05 | Canyon_Roadhouse | *Helminthoidichnites* | Nasep | Witputs |
| 2017-CR-1.06 | Canyon_Roadhouse | *Helminthoidichnites* | Nasep | Witputs |
| 2017-CR-1.10 | Canyon_Roadhouse | *Helminthoidichnites* | Nasep | Witputs |
| 2017-CR-1.11 | Canyon_Roadhouse | Plug-shaped | Nasep | Witputs |
| 2017-CR-1.12 | Canyon_Roadhouse | Plug-shaped | Nasep | Witputs |
| 2017-CR-1.24 | Canyon_Roadhouse | *Treptichnus* | Nasep | Witputs |
| 2017-CR-1.32 | Canyon_Roadhouse | *Helminthoidichnites* | Nasep | Witputs |
| 2017-CR-1.33 | Canyon_Roadhouse | *Treptichnus* | Nasep | Witputs |
| C16-SP-3.1 | Swartpunt | *Helminthoidichnites* | Spitzkop | Witputs |
| HB-10.1-2016 | Hansburg | Plug-shaped | Kliphoek | Witputs |
| LUCKY-2016 | Kuibis | Plug-shaped | Kliphoek | Witputs |
| TB-16-1.15 | Swartpunt | *Treptichnus* | Spitzkop | Witputs |
| TB-16-SP-1.10 | Swartpunt | *Planolites* | Spitzkop | Witputs |
| TB-16-SP-1.11 | Swartpunt | *Planolites* | Spitzkop | Witputs |
| TB-16-SP-1.12 | Swartpunt | Plug-shaped, *Treptichnus* | Spitzkop | Witputs |
| TB-16-SP-1.16 | Swartpunt | *Helminthopsis* | Spitzkop | Witputs |
| TB-16-SP-1.5 | Swartpunt | Plug-shaped | Spitzkop | Witputs |
| TB-16-SP-x.1 | Swartpunt | Plug-shaped | Spitzkop | Witputs |
| TB-SP-1.24 | Swartpunt | *Helminthoidichnites* | Spitzkop | Witputs |
| VC-1.6-2015 | Sontaagsbrun | *Treptichnus* | Nomtsas | Witputs |
| VC-2015-1.2 | Sontaagsbrun | *Treptichnus* | Nomtsas | Witputs |
| VC-2015-1.x | Sontaagsbrun | Treptichnus | Nomtsas | Witputs |

**S3.** Graphs indicating pairing between plug-shaped burrows. Despite no evidence for being linked in the subsurface, plug-shaped burrows are consistently found to be regularly spaced at 4 – 5 mm apart. A Shapiro-Wilk test for normalcy yielded a p-value of less than 0.05 (p = 0.0000915) for the distribution of nearest-neighbor distances. This spatial pattern was even more commonly observed when samples only contained a few pairs, although regular spacing was still common for samples with clustered burrows. Diameter size and distance to nearest neighbor are weakly positively correlated. A linear regression fit through the data has an R^2^ value of 0.183 and a Spearman’s Rank test yielded a 𝜌 value of 0.431 (p=0.00113).

**S4.** Point-counted bedding-plane bioturbation percentage results for slabs which were large enough to be point-counted.

| Trace fossil slab sample name | Stratigraphic member/formation | Percent of bedding-plane bioturbated (point-counted) |
| --- | --- | --- |
| 2017-AR-1.04 | Nasep | 3.24 |
| 2017-AR-1.15 | Nasep | 3.53 |
| 2016-BH-1.13 | Vingerbreek | 1.13 |
| 2016-BH-1.14 | Vingerbreek | 1.80 |
| 2017-CR-1.05 | Nasep | 0.93 |
| 2017-CR-1.12 | Nasep | 1.17 |
| 2017-CR-1.24 | Nasep | 2.43 |
| 2017-CR-1.33 | Nasep | 2.57 |
| 2017-CR-1.10 | Nasep | 4.15 |
| 2017-CR-1.04 | Nasep | 4.59 |
| 2017-CR-1.06 | Nasep | 4.66 |
| 2017-CR-1.32 | Nasep | 6.60 |
| 2016-HB-11.1 | Kliphoek | 1.00 |
| HB-10.1-2016 | Kliphoek | 2.42 |
| 2016-HH-1.1 b | Vingerbreek | 0.53 |
| 2016-HH-1.1 a | Vingerbreek | 0.59 |
| 2016-HH-3.4 | Vingerbreek | 0.76 |
| 2016-HH-1.2 | Vingerbreek | 1.03 |
| 2016-HH-1.15 | Vingerbreek | 1.38 |
| 2016-HH-1.8 | Vingerbreek | 3.22 |
| 17-FR-2.3 | Spitzkop | 0.72 |
| 17-FR-1.04 | Spitzkop | 5.43 |
| 17-FR-CK-3 | Spitzkop | 7.26 |
| 17-FR-1.01 | Spitzkop | 8.15 |
| 17-FR-CK-5 | Spitzkop | 8.15 |
| 17-FR-CK-1 | Spitzkop | 9.96 |
| 17-FR-1.07 | Spitzkop | 11.33 |
| 17-FR-CK-2 | Spitzkop | 12.64 |
| 17-FR-CK-4 | Spitzkop | 16.94 |
| 2016-DD-1.1 | Kliphoek | 0.20 |
| 2016-DD-1.2 | Kliphoek | 0.38 |
| 2016-DD-1.5 | Kliphoek | 1.06 |
| LUCKY-2016 | Kliphoek | 6.59 |
| 2016-NR-1.1 | Neiderhagen | 1.08 |
| 2016-SB-float-pedum | Nomtsas | 6.39 |
| VC-2015-1.x | Nomtsas | 10.06 |
| VC-2015-1.2 | Nomtsas | 17.94 |
| 2016-SB-1.31 | Nomtsas | 20.14 |
| 2016-SB-2.31 | Nomtsas | 25.48 |
| 2016-KK-1.4 | Vingerbreek | 0.72 |
| TB-16-SP-1.11 | Spitzkop | 0.97 |
| C16-SP-3.1 | Spitzkop | 1.69 |
| TB-16-SP-1.12 | Spitzkop | 1.69 |
| TB-16-SP-1.12 | Spitzkop | 1.69 |
| TB-16-SP-1.5 | Spitzkop | 1.74 |
| TB-16-SP-x.1 | Spitzkop | 2.25 |
| TB-16-SP-1.10 | Spitzkop | 3.43 |
| TB-16-SP-1.16 | Spitzkop | 3.58 |
| TB-16-1.15 | Spitzkop | 4.47 |
| TB-SP-1.24 | Spitzkop | 4.54 |
| 2016-US-10.1 | Urusis | 2.83 |

**S5.** Assignments and scores for EEI values. Tiering was assigned based on depth observed for trace fossils in this dataset. Functional group and bioirrigation potential was assigned by referencing the same or similar trace fossils in Herringshaw et al. (2017) and/or Minter et al. (2017).

| Ichnogenera | Depth | Functional group | Bioirrigation | EEI score |
| --- | --- | --- | --- | --- |
| *Helminthopsis* | Surficial (1) | Epifaunal locomotion (1), surficial modifiers (2) | Improbable (1) | 3 – 4 |
| *Helminthoidichnites* | Surficial (1) | Epifaunal locomotion (1), surficial modifiers (2) | Improbable (1) | 3 – 4 |
| Plug-shaped burrows | semi-infaunal (2) | surficial modifiers (2), biodiffusive bioturbators (3) | improbable (1) to possible (2) | 5 – 7 |
| *Planolites* | semi-infaunal (2) to shallow infaunal (3) | surficial modifiers (2), biodiffusive bioturbators (3) | Improbable (1), possible (2) | 5 – 8 |
| *Parapsammichnites* | semi-infaunal (2) to shallow infaunal (3) | biodiffusive bioturbators (3) | Improbable (1), possible (2) | 6 – 7 |
| *Treptichnus* | semi-infaunal (2) to shallow infaunal (3) | surficial modifiers (2), gallery biodiffusion (6) | Possible (2), probable (3) | 6 – 12 |

**S6.** Assignments ecosystem engineering cubes. Tiering was assigned based on depth observed for trace fossils in this dataset. Sediment modification and sediment interaction was assigned by referencing the same or similar trace fossils in Minter et al. (2017).

| Ichnogenera | Tiering | Sediment interaction | Sediment modification |
| --- | --- | --- | --- |
| *Helminthopsis* | semi-infaunal | backfill | conveyor |
| *Helminthoidichnites* | semi-infaunal | backfill | conveyor |
| Plug-shaped burrows | semi-infaunal | compression | gallery biodiffusion |
| *Planolites* | semi-infaunal | backfill | conveyor |
| *Parapsammichnites* | semi-infaunal | backfill | conveyor |
| *Treptichnus* | semi-infaunal | compression | gallery biodiffusion |

**S7.** Evidence of directed transport. A) Cross-bedding from a trace-fossil bearing bed at the Canyon Roadhouse locality in the Nasep. B) Asymmetrical ripple marks with plug-shaped burrows throughout from Farm Haruchas in the Vingerbreek. Upper red arrow indicates plug-shaped burrow in trough of ripple. Lower red arrow indicates plug-shaped burrow on peak of ripple. C) Tool marks from Koelkrans locality in the Spitzkop. Red arrow indicates *Planolites* trace fossil. White arrows indicate approximated direction flow estimated from tool marks on slab. D-E) *Helminthoidichnites* and sinsudoidal cresent ripple marks from Sontaagsbrun in the Nomtsas.


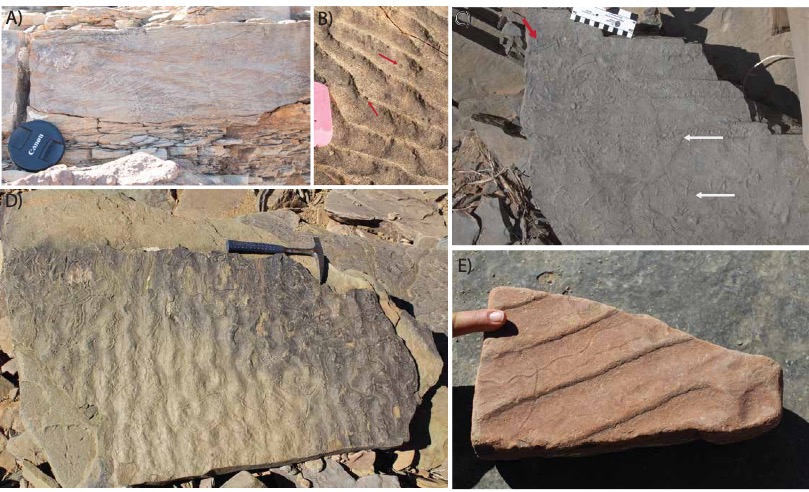


**Supplemental References**

1. Darroch, S. A. F., Boag, T. H., Racicot, R. A., Tweedt, S., Mason, S. J., Erwin, D. H., Laflamme, M. 2016. A mixed Ediacaran-metazoan assemblage from the Zari Sub-basin, Namibia. *Palaeogeography, Palaeoclimatology, Palaeoecology* **459**, 198-208. (doi:10.1016/j.palaeo.2016.07.003)

2. Mata, S. A., Corsetti, C. L., Corsetti, F. A., Awramik, S. M., Bottjer, D. J. 2012. Lower Cambrian anemone burrows from the upper member of the Wood Canyon Formation, Death Valley region, United States: Paleoecological and paleoenvironmental significance. *Palaios* **27**, 294-606. (doi: 10.2110/palo.2012.p12-016r)

3. Bouougri, E. H., Porada, H. 2007. Siliciclastic biolaminates indicative of widespread microbial mats in the Neoproterozoic Nama Group of Namibia. *Journal of African Earth Sciences* **48**, 38-48. (doi: 10.1016/j.jafrearsci.2007.03.004)

4. Seilacher, A., Buatois, L. A., Mángano, M. G. 2005. Trace fossils in the Ediacaran-Cambrian transition: Behavioral diversification, ecological turnover and environmental shift. *Palaeogeography, Palaeoclimatology, Palaeoecology* **227**, 323-356. (doi: 10.1016/j.palaeo.2005.06.003)

5. Carbone C, Narbonne GM. 2014 When life got smart: the evolution of behavioral complexity through the Ediacaran and Cambrian of NW Canada. *Journal of Paleontology* **88**, 309 – 330. (doi:10.1666/13-066)

6. Tarhan, L. G., Droser, M. L., Gehling, J. G., Dzaugis, M. P. 2017. Microbial mat sandwiches and other anactualistic sedimentary features of the Ediacara member (Rawnsley Quartzite, South Australia): Implications for interpretation of the Ediacaran sedimentary record. *Palaios* **32**, 181-194. (doi:10.2110/palo.2016.060)

7. Heer, O. 1877. Flora fossilis Helvetiae. Die Vorwletiche Flora der Schweiz. Zürich. p 182.

8. Buatois, L A., Almond, J., Mángano, M. G., Jensen, S., Germs, G. J. B. 2018. Sediment disturbance by Ediacaran bulldozers and the roots of the Cambrian explosion. *Scientific Reports* **8**, 4514. (doi: 10.1038/s41598-018-22859-9)

9. Nicholson, H. A. 1873. III. Contributions to the study of the errant Annelides of the older palæozoic rocks. *Proceedings of the Royal Society of London* **21**, 288-290.

10. Jensen, S., Runnegar, B. N. 2005. A complex trace fossil from the Spitzkop Member (terminal Ediacaran-? Lower Cambrian) of southern Namibia. *Geological Magazine* **142**, 561-569. (doi:10.1017/S00167568050000853)

11. Grotzinger JP, Bowring BZ, Saylor BZ, Kaufman AJ. 1995 Biostratigraphic and geochronologic constraints on early animal evolution. *Science* **270**, 598– 604. (doi:10.1126/science.270.5236.598)

12. Miller, S. A. 1889. North American geology and paleontology for the use of amateurs, students, and scientists: Cincinnati, Ohio: Western Methodist Book Concern.

13. Wilson J.P., Grotzinger, J. P., Fischer, W. W., Hand, K. P., Jensen, S., Knoll, A. H., Abelson, J., Metz, J. M., McLoughlin, N., Cohen, P. A., Tice, M. M. 2012 Deep-water incised valley deposits at the Ediacaran – Cambrian boundary in southern Namibia contain abundant *Treptichnus pedum*. *Palaios* **27**, 252– 273. (doi:10.2110/palo. 2011.p11-036r)

14. Bromley, R. G. 1996. Ichnotaxonomy and classification, ch.8. In *Trace Fossils: Biology, Taphonomy, and Applications*. London, UK: Chapman and Hall.

15. Jensen, S., Saylor, B Z., Gehling, J. B., Germs, G. J. B. 2000. Complex trace fossils form the terminal Proterozoic Namibia. *Geology* **28**, 143-146. (doi:10.1016/S0301-9268(99)00069-8)

16. Herringshaw, L. G., Callow, R. T., McIlroy, D. 2017. Engineering the Cambrian explosion: The earliest bioturbators as ecosystem engineers. *Geological Society, London, Special Publications* **448**, SP448-18. (doi:10.1144/SP448.18)

17. Minter, N. J., Buatois, L. A. Mángano, M. G., Davies, N. S., Gibling, M. R., MacNaughton, R. B., Labandeira, C. C. 2017. Early bursts of diversification defined the faunal colonization of land. *Nature Ecology & Evolution* **1**, 0175. (doi: 10.1038/s41559-017-0175)
